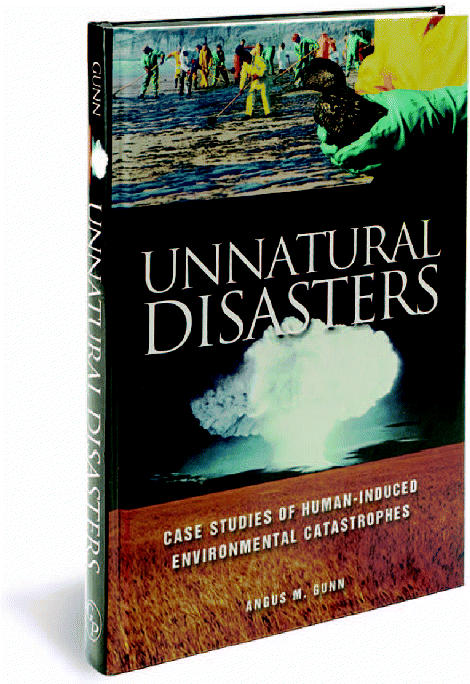# Unnatural Disasters: Case Studies of Human-Induced Environmental Catastrophes

**Published:** 2004-09

**Authors:** Johan M. Havenaar

**Affiliations:** Johan M. Havenaar, a psychiatrist, is director of the residency training program at Altrecht Institute for Mental Health Care in Utrecht, the Netherlands. He has published on the psychological consequences of human-made disasters, especially the Chernobyl disaster in Ukraine in 1986.

Angus M. Gunn

Westport, CT: Greenwood Press, 2004. 143 pp. ISBN: 0-313-31999-5, $55 cloth.

This book was written as a sequel to an earlier volume by the same author on the impact of natural disasters, *The Impact of Geology on the United States: A Reference Guide to Benefits and Hazards* (Westport, CT: Greenwood Press, 2001)*.* In this second volume, Angus M. Gunn provides an overview of human-made environmental disasters. He shows that although technology has given humankind enormous control over the environment, it has also proven to be a threat to our survival. Gunn categorizes these human-made disasters into a number of subtypes—for example, mining disasters, dam failures, government actions, industrial explosions, oil spills, nuclear energy catastrophes, and terrorism. For each of these types of disaster, the book contains 26 case examples describing the events that led up to the disaster, the technical details of the event itself, the cleanup it necessitated, and its consequences. Some of the examples described in the book are famous—for example, the Minimata mercury poisoning in Japan, the Buffalo Creek dam collapse in West Virginia, and the near accident at the Three Mile Island nuclear plant in Pennsylvania. Others have been almost forgotten, such as the deliberately induced great famine in Ukraine in 1932, which resulted from the massive collectivization of farms ordered by Stalin.

The book is well written and successfully combines factual information with good journalism. Gunn professes to stick to the tried-and-true methods of the hard physical sciences. The consequence of this choice is that the book makes no reference to the societal and psychological impacts of disasters. Interestingly, it is exactly some of the case examples given in the book, such as the Three Mile Island incident, that have led to the recognition of the importance of these secondary effects. This omission is especially obvious in the case of terrorist events, which are precisely intended to cause fear and social discord as much as physical damage. Readers who are interested in the full picture of the impact of human-made disasters, including their underlying psychological and societal dynamics, should therefore turn to other volumes (e.g., Havenaar JM, Cwikel JG, Bromet EJ, eds. *Toxic Turmoil: Psychological and Societal Consequences of Ecological Disasters.* New York:Kluwer Academic, 2002). Another shortcoming of the book is that some of the medical information cited in the book appears to be incorrect, as presented in the case of the Chernobyl accident. Without due reference the author states that this accident caused a steady rise in miscarriages and birth defects in Belarus and will eventually have generated a death toll of over 5 million people. Claims such as these are entirely unfounded, as noted by Bard et al. [Chernobyl, 10 years after: health consequences. Am J Epidemiol 19:1–18 (1997)].

In summary, *Unnatural Disasters* is a well-written book containing a wealth of historical details about some classical environmental disasters, but it is not suitable as a reference for public health purposes.

## Figures and Tables

**Figure:**